# Electric Field Measurement in Radiative Hyperthermia Applications

**DOI:** 10.3390/s25144392

**Published:** 2025-07-14

**Authors:** Marco Di Cristofano, Luca Lalli, Giorgia Paglialunga, Marta Cavagnaro

**Affiliations:** Department of Information Engineering, Electronic and Telecommunications, University of Rome La Sapienza, Via Eudossiana 18, 00184 Rome, Italy; marco.dicristofano@uniroma1.it (M.D.C.); lalli.1894733@studenti.uniroma1.it (L.L.); paglialunga.1839903@studenti.uniroma1.it (G.P.)

**Keywords:** hyperthermia, electric field measurements, electric field sensors

## Abstract

Oncological hyperthermia (HT) is a medical technique aimed at heating a specific region of the human body containing a tumour. The heat makes the tumour cells more sensitive to the cytotoxic effects of radiotherapy and chemotherapy. Electromagnetic (EM) HT devices radiate a single-frequency EM field that induces a temperature increase in the treated region of the body. The typical radiative HT frequencies are between 60 and 150 MHz for deep HT applications, while 434 MHz and 915 MHz are used for superficial HT. The input EM power can reach up to 2000 W in deep HT and 250 W in superficial applications, and the E-field should be linearly polarized. This study proposes the development and use of E-field sensors to measure the distribution and evaluate the polarization of the E-field radiated by HT devices inside equivalent phantoms. This information is fundamental for the validation and assessment of HT systems. The sensor is constituted by three mutually orthogonal probes. Each probe is composed of a dipole, a diode, and a high-impedance transmission line. The fundamental difference in the operability of this sensor with respect to the standard E-field square-law detectors lies in the high-power values of the considered EM sources. Numerical analyses were performed to optimize the design of the E-field sensor in the whole radiative HT frequency range and to characterize the sensor behaviour at the power levels of HT. Then the sensor was realized, and measurements were carried out to evaluate the E-field radiated by commercial HT systems. The results show the suitability of the developed sensor to measure the E-field radiated by HT applicators. Additionally, in the measured devices, the linear polarization is evidenced. Accordingly, the work shows that in these devices, a single probe can be used to completely characterize the field distribution.

## 1. Introduction

Oncological hyperthermia (HT) is a therapeutic technique used as an adjuvant to other cancer therapies (radio/chemotherapy) [1]. The aim of HT is to provide a heat source to increase the temperature of the tumour up to 40–44 °C for 60 min [2]. The temperature increase makes the tumour cells more permeable and sensitive to the cytotoxic effects of ionizing radiation and chemotherapy [3,4,5,6,7] and inhibits cancer cells from repairing DNA damage [8]. In the electromagnetic (EM) HT applications discussed in this paper, the heat source is an electromagnetic field radiated by applicators located on the surface of the body. The temperature increase is due to the absorption of the EM power by the treated region of the body [9,10,11].

HT applicators can be capacitive or radiative: the main differences among them are the working frequencies, applicator types, and direction of E-field polarization [12]. Capacitive devices operate at low frequencies, around 10 MHz [13]. Their applicators consist of two electrodes positioned on the opposite sides of the human body [13]. In this way, the electric field (E-field) is polarized orthogonally to the cranio-caudal axis of the human body. Some evidence showed that this aspect leads to an accumulation of heat in the subcutaneous fat layer [14]. Radiative HT applicators radiate an E-Field polarized parallel to the cranio-caudal axis of the human body [9]. They are made by antennas or antenna arrays that work in a vast frequency range, treating both deep and superficial tumours [9]. Deep hyperthermia (dHT) is performed in the frequency range 60–150 MHz, using power values up to 2000 W, while the current commercial superficial hyperthermia (sHT) applicators work at 434 MHz and 915 MHz with power values up to 250 W [9,15]. Since the penetration of EM energy is strongly dependent on frequency and the power deposition decreases at increasing depth into the tissue, sHT antennas aim at a therapeutic depth of 3–4 cm, while dHT antennas aim to reach the inner organs of patients of any size [9,15,16,17]. The Pennes’ bio-heat equation explains the relation between the temperature increase and the specific absorption rate (SAR), defined as the electromagnetic power absorbed per unit mass of tissue [18]. SAR depends on the dielectric properties of the tissues (relative permittivity ε_r_ and electric conductivity σ [S/m]). The temperature increase also depends on the thermal properties of the tissues (thermal conductivity [W/m·k] and specific heat capacity [J/kg·K]) and blood perfusion. Also, the metabolic heat generation influences the temperature distribution.

To provide uniform execution of clinical hyperthermia treatments and validate HT devices, quality assurance (QA) guidelines are essential. The current guidelines for deep [16] and superficial [15] hyperthermia are temperature-based, proposing thermal parameters for the characterization of the performance of hyperthermia devices [15,16]. These QA guidelines ask for specific equipment (e.g., a thermal imaging camera, a layered phantom) and careful procedures. However, the thermal parameters of the QA phantom have not been defined, and this leads to uncertainties in the evaluation of the QA parameters [19]. Besides temperature, the measurement of the electric field would allow assessment of the actual capability of a radiative HT applicator to generate the electromagnetic field within a phantom that simulates the dielectric behaviour of the human body. The E-field measurement could also be a key tool to verify if the device is able to generate the same EM field distribution over time (periodic checks), as well as throughout the time of an HT treatment, keeping the high-power values for about 1 h. Additionally, in dHT applications, the E-field distribution would allow verifying if the device is able to shift the position of the electric field focus according to the tumour location. Furthermore, in radiative HT, the linear polarization of the E-field is a crucial requirement that should be verified [15].

In this work, the development and use of an isotropic E-field sensor is proposed for measuring the E-field radiated by commercial HT applicators inside tissue equivalent phantoms. The sensor is proposed to be used during the validation stage of HT devices, before they are made available for clinical use, and to evaluate its performance after a certain period of use in the clinic. The typical structure of an E-Field sensor used for measurements inside a lossy medium is made by a linear dipole antenna, a non-linear detector, a high resistive transmission line, and the monitoring instrument [20,21]. Since the sensor detects the E-field component parallel to the dipole, three orthogonal sensors are needed to fully characterize an unknown electric field [21,22].

E-field sensors have been developed to measure the E-field induced in the human body by low-power devices, such as cellular phones [23], at mobile communication frequencies [24,25]. Accordingly, they typically operate in the square law range of the diode [23]. On the contrary, in HT applications, it is fundamental to prove the devices’ radiation capabilities when the typical clinical power values are used, to assess reproducibility, linearity, and stability with time of the HT devices. To this end, the feasibility of the use of the E-field sensor should be proven, characterizing the behaviour of the sensor response under the high-power radiation typical of HT devices. The sensor proposed in this work maintains the typical structure of electric field detectors, but it was optimized to work in the frequency range (60–915 MHz) and at the power levels typical of radiative hyperthermia (up to 2000 W). Furthermore, the sensor is proposed to be used for long time intervals, i.e., at least the entire duration of the HT treatment, to assess the device’s capability of maintaining its performance.

A method for qualitatively visualizing the HT electric field distribution based on the use of an LED matrix put inside a phantom was proposed in the literature [26,27]. The sensors in the matrix are dipoles connected to LED diodes: the light intensity of each LED depends on the electric field strength. The use of such matrices allowed a 2 cm spatial resolution in the plane orthogonal to the main E-field component and did not allow measurement of the three electric field components.

The E-field sensor developed in this work is composed of three probes disposed mutually orthogonal along the three spatial axes to detect the three E-field components. These are independently recorded to allow for evaluating the field polarization [28]. A numerical analysis was performed first to look for the optimal design and characterize the sensor behaviour for radiative HT. Two sensors were designed: one optimized for deep HT frequencies and the other optimized for superficial HT frequencies, respectively. They were then realized, and experimental measurements were conducted on a dHT and an sHT device. The measurement results show the suitability of the sensors to measure the E-field radiated by the two devices. They also demonstrated that the E-field is linearly polarized, so that the measurement of the main E-field component is sufficient to validate the considered HT devices. If the linear polarization is demonstrated, a single-probe sensor could be used to measure the E-field distribution with a spatial sampling step equal to at least the radius of the sensor (<4 mm) in the plane orthogonal to the main E-field component.

## 2. Materials and Methods

The E-field sensor is made by a short dipole with a diode connected between the dipole’s arms (Section 2.1). At first, simulations were carried out to characterize the optimal sensor design at the different working frequencies, considering the materials making the phantoms in which the sensors should be inserted (Section 2.2). EM simulations were performed to study dipoles with different dimensions, verifying the compliance with the short dipole condition, calculating the input impedance, and the effective length of the antenna (Section 2.2.1). Circuit simulations were then conducted to study the effect of the dipole’s length and the chosen diode on the sensor output voltage, and the working regimes of the sensor (Section 2.2.2). Then, the sensors were realized and used to measure the E-field distribution achieved in phantoms by two commercial hyperthermia devices (Section 2.3).

### 2.1. E-Field Sensor’s Structure

The typical structure of an E-field sensor proposed in literature is composed by a detector integrated in a short dipole. In [29], the short dipole condition is defined: the dipole length must be ≤λ/10, where λ is the wavelength at the working frequency in the considered material. If the short dipole condition is met, the input impedance of the dipole is purely capacitive, and its effective length is approximately half its length [21].

**Figure 1:**
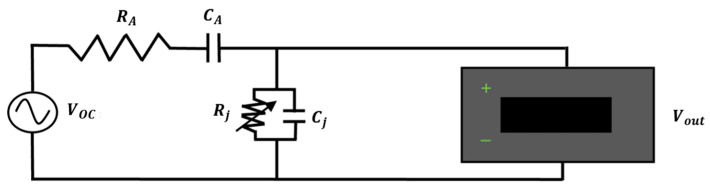
Equivalent circuit of the E-field sensor.

The E-field sensor realized in this work is composed by three probes mutually orthogonal: each probe consists of a Schottky diode soldered between the arms of a short dipole antenna. The electric field component parallel to the dipole ($E_{inc,co-pol}$) induces a sinusoidal open-circuit voltage $\left(V_{OC}\right)$ on the antenna gap given by the following relationship [21]:

(1) $$V_{OC}=h_{e}\cdot E_{inc,co-pol}$$

where $h_{e}$ is the effective length of the dipole. The open-circuit voltage is rectified by the diode, giving rise to different frequency components. The high impedance lossy transmission line that connects the diode to the reading unit cuts the sinusoidal components, allowing only the DC component to be detected [20]. The sensor equivalent circuit is shown in Figure 1 [21,22]. In Figure 1, the dipole antenna is represented by the equivalent voltage generator ($V_{OC}$—Equation (1)) and by its impedance shown as the series of a resistance ($R_{A}$) and a capacitance ($C_{A}$). However, as already reported, for a short dipole antenna the resistance is negligible compared to the impedance associated with the capacitance; consequently, the short dipole impedance can be represented only by its capacitance. In Figure 1, the diode is represented by a parallel between the junction resistance ($R_{j}$) and the junction capacitance ($C_{j}$) [21]. The output voltage ($V_{out}$) depends on $V_{OC}$, the dipole antenna’s impedance (${Z_{A}=R}_{A}+\frac{1}{j\omega C_{A}}$) and the diode’s response. In the small signal regime, the link between the E-field amplitude and $V_{out}$ is quadratic, while, in the large-signal regime, the output voltage is linearly proportional to the amplitude of the E-field component parallel to the dipole [21,22].

### 2.2. Simulation Analysis

#### 2.2.1. Electromagnetic Simulations

The EM simulations were performed using CST Microwave Studio (2021 version, Dassault Systèmes, Vélizy-Villacoublay, France). The numerical analysis was conducted at the radiative hyperthermia frequencies (60–915 MHz).

The dipole was realized as a cylindrical perfect conductor of different lengths and with a radius of 0.3 mm. The studied lengths were 2, 3, 4, 5, and 6 cm to look for the best compromise among experimental feasibility, resolution, and sensitivity. The dipole was coated with two cylindrical layers of low-loss materials to isolate it from the surrounding medium. The inner coating layer was made by resin (ε_r_ = 3, tanδ = 0.0065 [30]; with an outer radius of 2.5 mm), while the second one was made by Teflon (ε_r_ = 2.1, tanδ = 0.0002 [CST material Library]; with an outer radius of 3.5 mm).

The dipole was immersed inside a lossy medium, modelled as a cube of 50 cm side, ensuring dimensions much larger than the dipole length. Two different muscle equivalent saline solutions, simulating the mixtures used in the experimental procedures, were used to fill the phantom. The dielectric properties values were set according to the measured values reported in Section 2.3.3.

**Figure 2:**
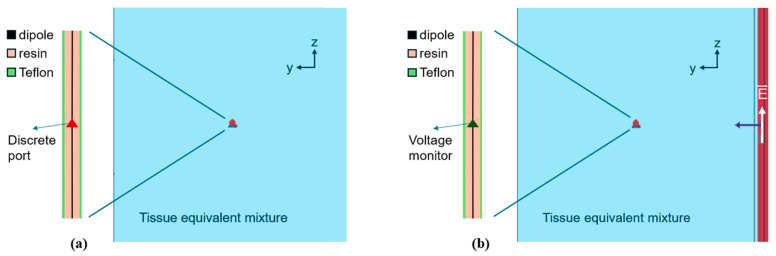
Simulation scenarios of the linear antenna inside the lossy medium: (**a**) dipole in transmitter configuration (discrete port) for the evaluation of the input impedance; (**b**) plane wave (red layer on the right of the figure with white arrow representing the electric field polarization) impinging on the dipole in receiver mode (voltage monitor) for the evaluation of the dipole’s effective length.

Two simulation scenarios were defined as shown in Figure 2. In the first scenario (Figure 2a), the dipole was simulated in transmitter configuration. The input impedance of the dipole was evaluated. The analysis aimed at assessing whether the dipole impedance remained purely capacitive at the studied frequencies for the coated dipole of the different lengths in the dissipative medium. In addition, the impedance values were recorded to be successively given as input parameters for the circuit simulations (see Section 2.2.2). In the second scenario (Figure 2b), the dipole was studied in receiver mode to obtain the dipole’s effective height. To this end, a plane electromagnetic wave with the propagation direction along the y axis (Figure 2b) and the electric field vector parallel to the dipole axis (z axis in Figure 2b) was introduced as the EM source. A voltage monitor was placed between the dipole arms to evaluate the open-circuit voltage ($V_{OC}$) induced by the E-field. Since the plane wave is propagating in a dissipative medium, to obtain the actual E-field value impinging on the dipole ($E_{inc,co-pol}$), an E-field probe was positioned at 1 mm distance from the dipole. The dipole effective length was then calculated using (1).

#### 2.2.2. Circuit Simulation

The sensor equivalent circuit (Figure 1) was simulated using NI Multisim (version 14.3, National Instruments, Austin, TX, USA) in the whole radiative hyperthermia frequency range (60–915 MHz). The numerical analysis aimed at defining the optimal design conditions of the dipole-diode configuration at deep and superficial HT frequencies. Furthermore, the circuit simulations allowed the characterization of the working regimes for the sensor according to the values of the E-field received by the dipole.

In the circuit simulations, at the studied frequencies, the dipole lengths that verified the short dipole condition, and therefore showed a purely capacitive impedance, were considered only. For this reason, in the circuit, $R_{A}$ was neglected and $Z_{A}\approx \frac{1}{j\omega C_{A}}$. The sensor circuit was simulated setting the input voltage (corresponding to the open-circuit voltage between the dipole’s arms—$V_{OC}$), the antenna capacitance as calculated with the EM simulations, the working frequency, the Schottky diode, and evaluating the sensor output voltage ($V_{out}$).

The numerical analysis was performed to characterize the sensor response ($V_{out}$/$V_{OC}$) for different values of the circuit parameters at the studied frequencies. When the effect of $C_{A}$ was studied, the values of $R_{j}$ and $C_{j}$ of the diode were fixed; when the effect of the diode on the sensor’s response was studied, $C_{A}$ was kept fixed. At RF frequencies, the behaviour of a Schottky diode is dominated by the junction capacitance rather than junction resistance [31]. Accordingly, the effect of the diode’s junction capacitance ($C_{j}$) was studied considering $C_{j}$ values in a wide range of commercial Schottky diodes, i.e., in the range 0.4–10 pF. To study the sensor’s working regime, the proportionality relation between the input ($V_{OC}$) and output ($V_{out}$) voltages was evaluated for increasing values of the input voltage. The $V_{OC}$ values at which the transition from quadratic to linear regime takes place were identified.

### 2.3. Experimental Analysis

#### 2.3.1. Choice of the Optimal Sensor Configuration

Following the results of the simulation analysis, two E-field sensors were realized, one optimized at dHT frequencies, the other at sHT frequencies. The length of the two dipoles was selected to find the trade-off between the maximization of the sensor’s response ($V_{out}/V_{OC}$) and an optimal spatial resolution. In fact, the dipole receives the electric field that impinges on its full length; hence, it integrates the electric field values along its length. The use of a shorter dipole allows a higher measurement spatial resolution but reduces the sensor’s sensitivity. The lengths of the probes and the diode junction capacitance used for the realization of the sensors are detailed in the Results section (Section 3). Furthermore, another parameter considered in the choice of the diode was the breakdown voltage. Once the optimal value of $C_{j}$ is defined, a high value of breakdown voltage is preferable.

#### 2.3.2. Realization of the E-Field Sensors

To realize the single probe, the copper rheophores of the Schottky diode were cut in order to operate as dipoles of the desired length. This resulted in the linear antenna with the integrated diode. Subsequently, two conductive thermoplastic polyurethane (TPU) cables (high impedance lossy line) were crimped onto the two opposite ends of the dipole, respectively. The other end of the TPU cables was connected to a digital multimeter (GDM-8341, GW INSTEK, Taipei, Taiwan) for the voltage reading. Each probe was then encapsulated inside the isolation layers: it was inserted inside a Teflon cylinder, obtained by the cap of a commercial syringe, which was then filled with the resin.

To realize an isotropic sensor, the above procedure was repeated for three probes, which were then fixed on a plastic support so as to be mutually orthogonal and arranged along the 3 spatial axes.

#### 2.3.3. Experimental Setup: Phantoms and Mixtures

Once realized, the E-field sensors were used to perform measurements on two commercial hyperthermia devices: a sHT applicator (β Antenna, ALBA ON 4000D, Medlogix Srl, Rome, Italy) working at 434 MHz, and a dHT system (ALBA 4D, Medlogix Srl, Rome, Italy) working at 70 MHz. The experimental measurements were performed by inserting the E-field sensors in a plexiglass phantom filled with muscle equivalent mixtures. The presence of the plexiglass layer, characterized by low relative permittivity and electric conductivity, simulates the fat-muscle interface. For the sHT measurement, a curved phantom was used to simulate the surface of the human body on which the antennas are located during the treatment. For the measurements at dHT frequencies, the section of the phantom had a dodecagonal shape in order to simulate the elliptical shape of the abdominal area of the human body. For both phantoms, the plexiglass structure was 3 mm thick. A big aperture to allow the placement of the sensor and its movement is present in the area of the phantoms that precedes the place where the HT devices were located.

As previously described, the phantoms were filled with two different saline solutions: a 6 g/L NaCl solution was used at 434 MHz, and a 3 g/L NaCl solution at 70 MHz. The amount of NaCl in the saline solution influences the conductivity: as the NaCl concentration increases, the electric conductivity increases [32]. The different amounts of NaCl are used to represent the muscle’s conductivity at sHT and dHT frequencies. The NaCl concentration in the mixtures was checked before each measurement using the EZ-9909 device (Filtronic, NETPark, County Durham, UK). The dielectric properties of the used mixtures were measured through the open-ended coaxial probe technique: the S_11_ generated at the interface between the open-ended coaxial probe’s tip and the mixture was measured using a VNA (E8363C PNA Microwave Network Analyzer, Keysight, Santa Rosa, CA, USA). Then, the dielectric properties were derived through mathematical models [33,34]. To derive the dielectric properties from the S_11_, the system was calibrated by measuring the S_11_ of three known terminations (short circuit, open circuit, distilled water). The measurement uncertainty calculated by applying the model for a liquid with known dielectric properties (0.9% saline solution) was lower than 10%. The measured dielectric properties (i.e., 3 g/L NaCl saline solution: ε_r_ = 83.2 and σ = 0.58 S/m at 70 MHz; 6 g/L NaCl saline solution: ε_r_ = 75.5 and σ = 1.07 S/m at 434 MHz) were set for the EM simulation analysis (Section 2.2.1). It is important to note that for the quantitative evaluation of the electric field radiated by hyperthermia devices, it is necessary for the dielectric properties of the used mixtures to be well representative of those of the muscle. It is worth noticing here that the work focuses on the characterization of the sensor at radiative HT working conditions, so that the phantom’s design was not optimized. Indeed, for the purpose of this work, any difference in the dielectric properties of the mixtures from those of the muscle has no impact on the comparison of the three E-field components, since these are measured under the same conditions.

#### 2.3.4. Measurements’ Procedure

During the measurements, the sensor was positioned on a plastic rod, it was immersed inside the solution, and moved inside the phantom through a robotic system. 

**Figure 3:**
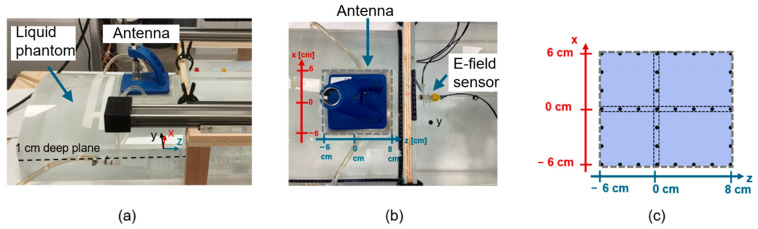
Experimental setup for measurements of E-field induced by a superficial HT device (ALBA ON 4000D, β antenna, Medlogix, Rome, Italy): (**a**) 3D view of the superficial HT applicator over the phantom with 3D reference system (**b**) top view; (**c**) measurement points inside the 1 cm deep xz plane.

For the experimental setup at sHT frequencies, the β antenna (Medlogix, Rome, Italy) was positioned over the phantom and connected to the RF generator through a coaxial cable. The used antenna belongs to the class of microstrip applicators, typically used for superficial HT [35,36,37]. The RF input power, controlled using a proprietary software (ALBA ON 4000D software, Medlogix, Rome, Italy), was set at 80 W. The isotropic sensor (optimized for sHT frequencies) was positioned on the robotic arm and immersed at 1 cm depth inside the phantom (Figure 3a). Measurements were taken by moving the sensor in the xz plane (Figure 3a). First, the sensor was located at three fixed z positions (z = −6 cm; 0 cm; 8 cm; Figure 3b,c) and moved along the x direction with 2 cm step. Then measurements were performed along the z direction with 2 cm step at three different fixed x positions (x = −6 cm; 0 cm; 6 cm Figure 3b,c). The velocity of the movement was set quite slow, to avoid moving the phantom liquid. In addition, once the sensor was in each new position, a time interval of at least 10 s was waited for before taking the first measure to allow the system to stabilize for any perturbation due to the sensor location. All the measurement points are shown in Figure 3c. Those points have been selected to cover all the areas below the antenna, including both the EM focus and potential boundary effects near the antenna’s margins.

**Figure 4:**
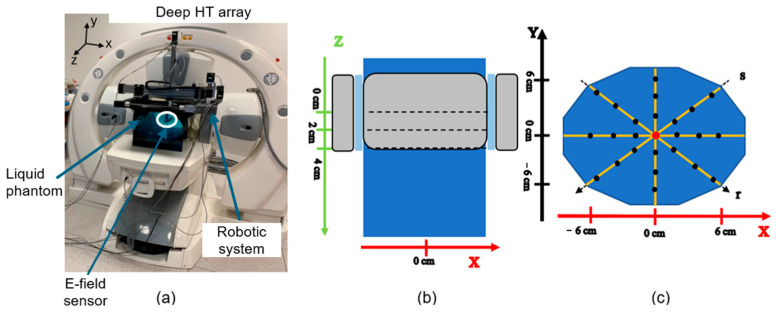
Experimental setup for deep HT frequency (ALBA 4D, Medlogix, Rome, Italy): (**a**) 3D arrangement; (**b**) top view of the experimental setup and studied z positions; (**c**) measurement points in the xy planes (at the different z levels).

In the setup used for the measurement with the ALBA 4D system, the E-field sensor optimized at deep HT frequencies was used. The ALBA 4D system (Figure 4a) is a phased array of 4 waveguide antennas working at 70 MHz [38,39,40]. A plexiglass phantom filled with the 3 g/L NaCl saline solution was positioned inside the applicator. The sensor was moved inside the phantom through the robotic system. The total input power was 600 W (150 W per antenna). The input phases of the antennas were chosen to have a constructive interference at the centre of the phantom (defined as reference point: x = 0 cm; y = 0 cm; z = 0 cm). The input power and the phases were set and checked using a proprietary software (ALBA 4D software, Medlogix, Rome, Italy). The measurements were made on three different xy planes (Figure 4b) of the phantom: z = 0 cm (central plane with respect to the applicator), z = 2 cm, z = 4 cm. For each section, different measurement points along 4 different directions (x, y, r, and s) were investigated, as shown in Figure 4c. The measurement points were selected to cover almost all the phantom’s transversal area and evaluate potential boundary effects. A spatial sampling step of 2 cm was used.

In both measurement scenarios, the three probes composing the isotropic sensor were connected simultaneously to three digital multimeter (GDM-8341, GW INSTEK, Taipei, Taiwan). The accuracy of the multimeter was $\delta_{m}=$ ± 0.02% of reading + 0.0004 V [41]. The background noise detected by the sensor was always of the order of 10^−3^ V in the measurement setup for sHT. In the setup for dHT, given the dimension of the system and the presence of the electronics required to power the 4 antennas, the background noise was higher. To minimize it, the setup was equipped with decoupling elements, such as ferrites applied to the transmission line connecting the sensor to the multimeter, and using wooden or plastic supports for positioning the measuring instrument. By implementing these precautions, the background noise was always of the order of 10^−2^ V. When the measurement was performed, before feeding the HT antennas, the background noise was measured in each measurement point; then, it was subtracted from the measured value before elaborating the results. The relative position between the antennas and the robotic system was marked and kept fixed, so it was possible to repeat the measurements five times for each studied direction, always considering the same measuring points. The mean value and the standard deviation of the detected voltage in the 5 measurements were then calculated and are reported in the results section (Section 3.3.1). All measurement results were normalized to the maximum output voltage measured along the studied direction. Since measured data showed that the component of the field co-polarized with the antenna (z direction in both setups) was greater than the other two components, the percentage variation between the measured voltage for the field component along this direction (V_z_) and the square root of the quadratic sum of the three measured voltages (V_tot_) was calculated. This calculation aimed at determining whether the other two components can be considered negligible. The same measurement procedure was repeated on 3 different days, giving results consistent with those shown in this work.

## 3. Results

### 3.1. Numerical Results

#### 3.1.1. Results of Electromagnetic Simulations

The EM simulations were focused on the study of the fulfilment of the short dipole condition in the studied application (coated dipoles inside a lossy medium), and the evaluation of the effective length of the dipoles. The ratio *l*/λ was calculated considering both the wavelength in the vacuum ($\lambda_{0}$) and the wavelength in the resin ($\lambda_{r}=\frac{\lambda_{0}}{\;\sqrt{\varepsilon_{r}}}$ [42], where $\varepsilon_{r}=3$ is the dielectric permittivity of the resin). Table 1 and Table 2 report the ratio $\frac{l}{\lambda }$ at different frequencies and different dipoles’ lengths calculated in the vacuum and the resin, respectively.

From the theory, the short dipole condition is no longer valid for dipoles longer than 4 cm at 915 MHz in vacuum. Considering the resin surrounding layer, the short dipole condition is no longer valid for dipoles longer than 4 cm at 434 MHz, and longer than 2 cm at 915 MHz. The short dipole condition is valid at deep HT frequencies for all the studied dipole lengths.

**Figure 5:**
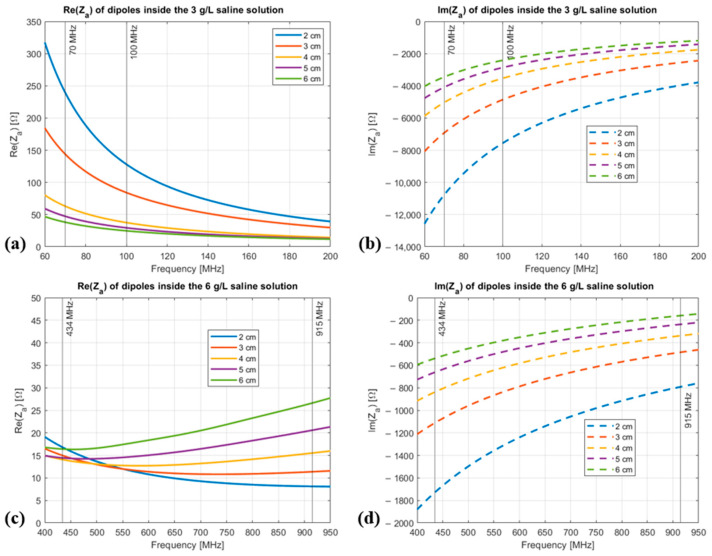
Frequency behaviour of the input impedance of dipoles of different lengths inside the saline solutions: (**a**) Re{Z_A_} inside the 3 g/L saline solution at dHT frequencies; (**b**) Im{Z_A_} inside the 3 g/L saline solution at dHT frequencies; (**c**) Re{Z_A_} inside the 6 g/L saline solution at sHT frequencies; (**d**) Im{Z_A_} inside the 6 g/L saline solution at sHT frequencies.

Figure 5 shows the frequency behaviour of the real (Re{Z_A_}) and imaginary (Im{Z_A_}) parts of the input impedance of dipoles of different lengths inside the saline solutions used for deep and superficial HT frequencies.

As shown in Figure 5, in the whole radiative HT frequency range (60–915 MHz) and for all considered dipole lengths, the imaginary part of the input impedance of the dipoles is much greater than the real part, so that it can be treated as purely capacitive.

Table 3 reports the effective length ($h_{e}$) of the dipoles derived from the EM simulations and calculated by (1) as described in Section 2.2. The results are reported only for dipoles that verified the short dipole condition (Table 2).

From the Table, it is derived that the effective length of the dipoles is close to half of their geometrical length. This result agrees with the literature [21].

#### 3.1.2. Circuit Simulations

**Figure 6:**
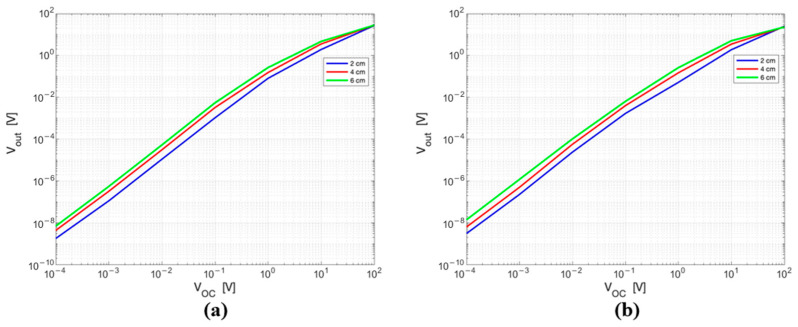
Equivalent circuit output voltage as a function of the input voltage for different lengths of the dipole at (**a**) 70 MHz and (**b**) 434 MHz. Both axes are in logarithmic scale.

The effect of the dipole length and diode’s junction capacitance on the output voltage was studied considering the whole radiative HT frequency range. Figure 6 displays the output voltage ($V_{out}$) as a function of the input voltage (*V_OC_*) for different lengths of the dipole at 70 MHz (Figure 6a) and at 434 MHz (Figure 6b). The different lengths are represented in the circuit by different $C_{A}$ values, i.e., those obtained from the EM simulations (Figure 5). The diode junction capacitance used in these simulations is 2 pF.

As shown in Figure 6, the sensor response ($V_{out}/V_{OC}$) increases as the dipole length increases, i.e., for decreasing values of the antenna capacitance. The effect defined by (1), i.e., that, as the dipole length increases, the $V_{OC}$ amplitude also increases, should also be added to this result. It is pertinent to note that the studies performed at the other frequencies produced congruent outcomes.

**Figure 7:**
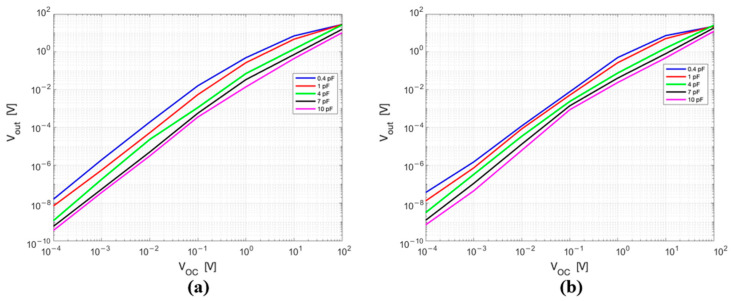
Equivalent circuit output voltage as a function of the input voltage for different diode’s junction capacitances at (**a**) 70 MHz and (**b**) at 434 MHz.

Figure 7 shows the output voltage ($V_{out}$) as a function of the input voltage ($V_{OC}$) for different diode’s junction capacitance values, for a fixed dipole length (6 cm). The considered $C_{j}$ values vary from 0.4 pF (which corresponds to BAT 62-03W (Infineon Technologies, Neubiberg, Germany) Schottky diode) to 10 pF (which corresponds to BAT 54 Schottky (Infineon Technologies) diode). In particular, Figure 7a reports the values at 70 MHz, and Figure 7b at 434 MHz. The two different frequencies are represented by the different values of the antenna input capacitance ($C_{A}$).

The sensor response ($V_{out}/V_{OC}$) increases as the diode’s junction capacitance decreases: the higher sensor response is obtained for the lowest value of $C_{j}$. From the graphs in Figure 6 and Figure 7, it is relevant to mention that the relationship between $V_{out}$ and $V_{OC}$ is quadratic until $V_{OC}$ is below 0.1 V: the circuit is working within the small signals’ region. When $V_{OC}$ is above 1 V the relationship becomes linear (large signal regime) [21]. Between these two regions—approximately in the range from 0.1 to 1 V—the diode operates in an intermediate regime, where the transition between the quadratic and linear behaviour gradually occurs.

### 3.2. Optimal Configuration and Realisation of the E-Field Sensors

Based on the results of the EM and circuit simulations and on the size requirements for the applications, two optimal configurations for the probes realization were chosen.

At 70 MHz, a probe’s configuration made by a 4 cm long dipole and a 2 pF diode’s junction capacitance was chosen. In fact, the longer the dipole, the higher its sensitivity. However, a 6 cm long dipole impacts the resolution of the probe, as introduced in Section 2.3.1. To meet the trade-off between sensor response and resolution, the 4 cm length was chosen. For the same reason, a 2 cm long dipole was selected at the frequency of 434 MHz. In this case, with a view to proposing a sensor that works at all sHT frequencies, the length < 3 cm was necessary to meet the short dipole condition at 915 MHz also. For both the frequencies in the exam, a Schottky diode with a 2 pF $C_{j}$ (1N5711, RF Microwaves, Cesano Maderno, Italy) was chosen. The Schottky diode 1N5711 represents the best compromise between the breakdown voltage (which shows the highest value, [i.e., 70 V]) and the junction capacitance (which has the smallest value, corresponding to the high sensitivity of the diode) among those available from different factories.

Considering the specifications described above, 3 probes for each of the two configurations were realized (following the fabrication process described in Section 2.2.2) and assembled to make the isotropic sensors.

**Figure 8:**
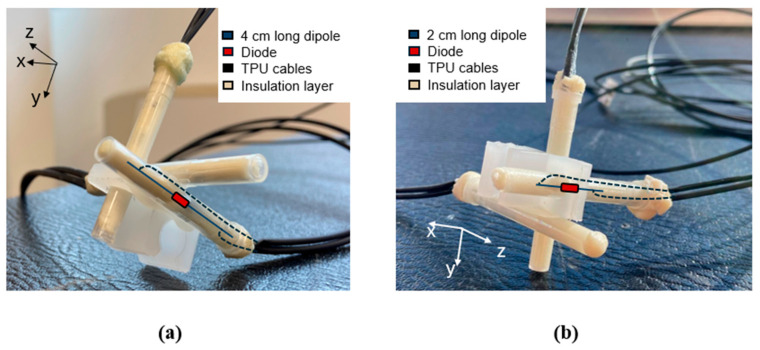
Realized isotropic E-field sensors to be used at 70 MHz (**a**) and 434 MHz (**b**).

The final structures of the E-field sensors are shown in Figure 8.

### 3.3. Measurements Results

In this section, the values detected by the isotropic E-field sensor induced into the phantoms by the ALBA ON 4000D (β antenna) and by ALBA 4D are reported. The results of all the E-field components are normalized to the maximum value of the quadratic sum of the three measured voltages (V_tot_).

#### 3.3.1. E-Field Measurements for Superficial HT

**Figure 9:**
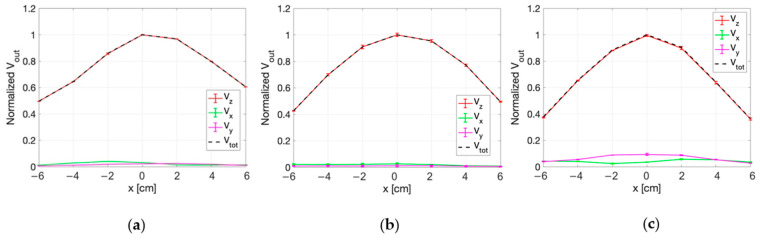
Normalized voltage detected by each probe of the isotropic E-field sensor as a function of x, at: (**a**) z = − 6 cm, (**b**) z = 0 cm, (**c**) z = 8 cm. The quadratic sum is also reported.

**Figure 10:**
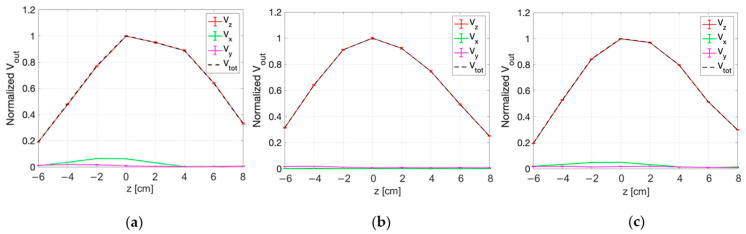
Normalized voltage detected by each probe of the isotropic E-field sensor as a function of z, at: (**a**) x = − 6 cm, (**b**) x = 0 cm, (**c**) x = 6 cm. The quadratic sum is also reported.

The mean values over the 5 repetitions of the signal measured by each probe along the x axis of the phantom (at the three fixed z positions) are reported in Figure 9. In the figure, vertical bars represent the standard deviation of the measurements. Figure 9 also reports the quadratic sum of the three measured voltages (V_tot_). Similarly, Figure 10 reports the same data measured along the z-axis for three different positions along the x-axis.

For all the measurements, the standard deviation was smaller than 5%. From the figures it can be appreciated how the probe oriented along the z-axis is the one who detects the more intense signal compared to the other two probes of the sensor.

Considering all the measurement points, the maximum value of the percentage variation between V_z_ and V_tot_ is 1.3%. This value demonstrates that the E-field components along the x and y directions are negligible with respect to the one along the z direction.

#### 3.3.2. E-Field Measurements for Deep HT

**Figure 11:**
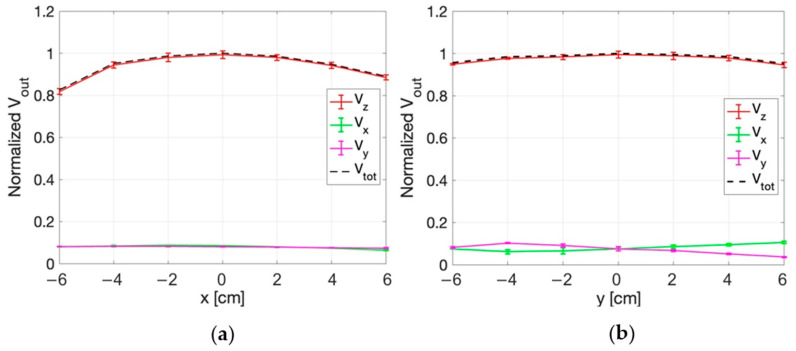
Normalized voltage detected by each probe of the isotropic E-field sensor and quadratic sum, as a function of x (a) and y (b) at z = 0 cm.

**Figure 12:**
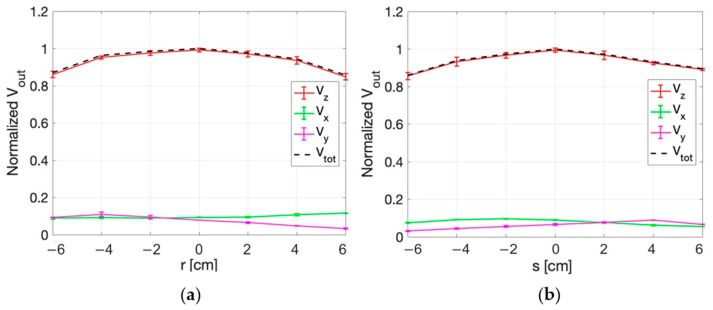
Normalized voltage detected by each probe of the isotropic E-field sensor and quadratic sum, along r (**a**) and s (**b**) spatial directions at z = 0 cm.

The measurement set-up for dHT applications is described in Section 2.2.2. Figure 11 and Figure 12 show the normalized values of the voltages detected by the three probes of the sensor along the x, y (Figure 11), r, and s directions (Figure 12) in correspondence with z = 0 cm. The figures report the standard deviations of the five repetitions of the measurements as vertical bars. The quadratic sum of the three measured voltages (V_tot_) is also shown.

From the figures it can be appreciated how the highest output voltage was detected by the probe oriented along the z-axis in all the measurement points.

**Figure 13:**
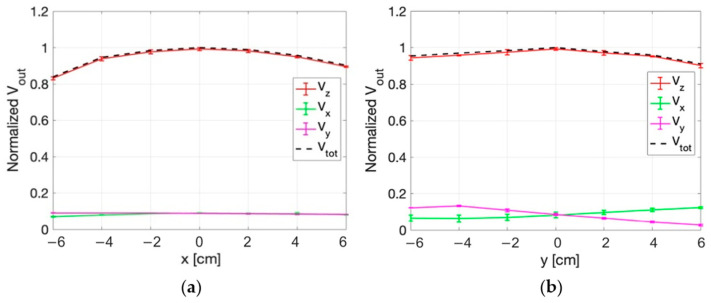
Normalized voltage detected by each probe of the isotropic E-field sensor and quadratic sum, as a function of x (**a**) and y (**b**) at z = 2 cm.

**Figure 14:**
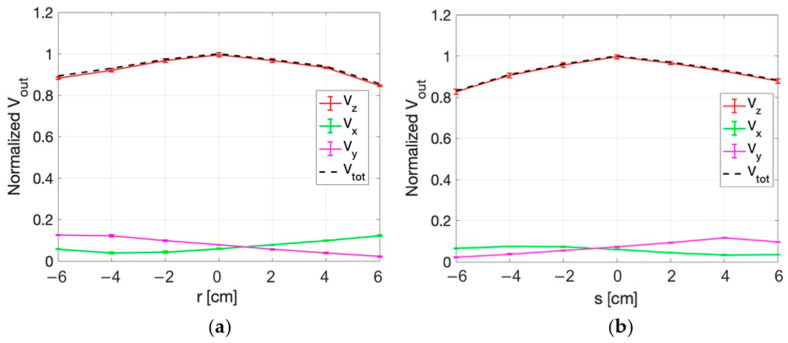
Normalized voltage detected by each probe of the isotropic E-field sensor and quadratic sum, along r (**a**) and s (**b**) directions at z = 2 cm.

**Figure 15:**
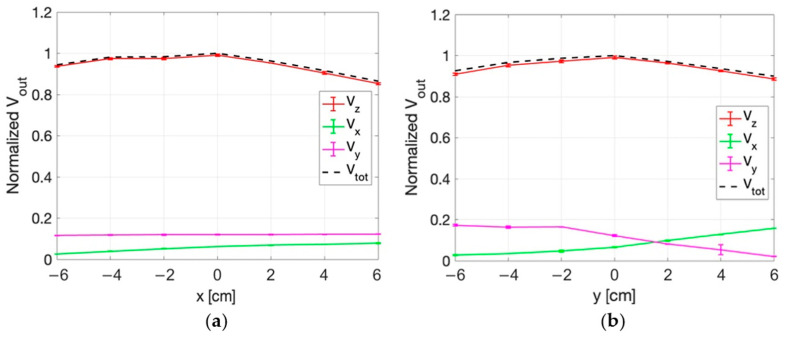
Normalized voltage detected by each probe of the isotropic E-field sensor and quadratic sum, as a function of x (**a**) and y (**b**) at z = 4 cm.

**Figure 16:**
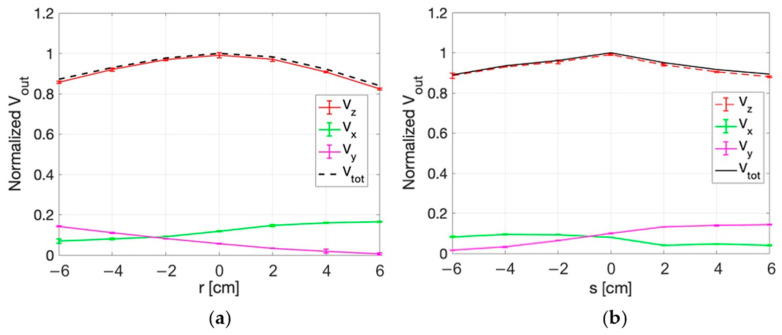
Normalized voltage detected by each probe of the isotropic E-field sensor and quadratic sum, along r (**a**) and s (**b**) directions at z = 4 cm.

Similar results are obtained in the planes at z = 2 cm and z = 4 cm, as reported in Figure 13, Figure 14, Figure 15 and Figure 16. An edge effect can be appreciated in correspondence of the external measurement points (edges of antennas) where the V_x_ and V_y_ voltages vary from the central values. This effect is more pronounced along the y direction and the two diagonals (r and s directions) compared to the x direction.

The standard deviation among the measurements was calculated and proved to be always smaller than 2%, as also evident from the small vertical bars in the figures.

Considering all the measurement points, the maximum percentage variation between V_z_ and V_tot_ is smaller than 2%. This result demonstrates that the x and y components of the E-field are negligible with respect to the z component.

## 4. Discussion

The current study proposes the development and use of an isotropic E-field sensor for the evaluation of the electric field distribution radiated by superficial and deep hyperthermia devices. The use of the isotropic E-field sensor allows for the characterization of the capabilities of the devices to radiate the requested electric field all along a treatment duration, to verify focusing capabilities, as well as to evaluate the actual capability of the devices to radiate a linearly polarized electric field throughout the region of interest. In fact, for radiative HT, the E-field should be linearly polarized along the cranio-caudal axis of the body [9,33,34,35,36,37,38]. The developed sensor could be used as part of the validation procedures and periodic performance assessments for radiative HT devices.

First, a numerical characterization of an E-field sensor composed by a short dipole, a Schottky diode, a high impedance transmission line, and a monitoring instrument was performed. The results of the EM and circuit simulations showed optimal dipole lengths of 2 cm for superficial HT and 4 cm for deep HT, with lower diode capacitance enhancing sensitivity. The numerical analysis also allowed to identify the working regimes of the sensor. For open-circuit voltage ($V_{OC}$) values below 0.1 V, the sensor operates in the quadratic regime, while for $V_{OC}$ values above 1 V, the operating regime is linear. For $V_{OC}$ values between 0.1 V and 1 V, an intermediate transition regime is identified.

E-field sensors made by a linear dipole loaded with a diode were developed to measure the SAR induced inside lossy media by mobile communication systems [43], that are low-power devices. Low-power applications allow such sensors to act as square-law detectors. As reported above, in this work, the sensor’s response was characterized considering broader power levels to represent the values typical of radiative hyperthermia (10 W–2 kW) and the corresponding frequency range (60–915 MHz).

An additional point to notice is that since the proposed sensor employs a short dipole antenna, the interference with HT antennas is minimized. Indeed, experiments confirmed the absence of impact on the HT antennas’ matching or the power radiated by both sHT and dHT systems. With reference to the effect of the presence of the sensor on the field distribution, preliminary simulations showed that the effect on the field is constrained within a few mm around the transversal section of the dipole and all along the sensor length. Such a volume represents the integration volume of the sensor, that determines its response. These preliminary results are also compliant with literature data (e.g., [44]).

Once the sensors have been realized, measurements in tissue-equivalent phantoms characterized the three components of the electric field radiated by an sHT and a dHT device. The results confirmed that the studied devices radiate an electric field linearly polarized along the cranio-caudal direction of the human body. Therefore, the use of a sensor composed of a single probe parallel to the main E-field component is sufficient to characterize the electric field distribution radiated by these devices. Besides the E-field polarization, the measurements can also be used to identify the position of the EM field focus generated by the HT device under study. In fact, the position of the E-field focus can be detected as the position where the maximum value of the sensor response is measured.

In the literature, with reference to HT applications, electric field measurements were performed using LED matrices [26,27]. With respect to these, the E-field sensor proposed in this work shows a much better resolution [45] and, having realized an isotropic probe, allows the measurement and analysis of the three E-field components. Indeed, the LED matrix allows the measurement of the cranio-caudal co-polarized component only. With reference to the devices measured in this work, the cranio-caudal co-polarized component was found to be the dominant one, so that a single, linear probe can be used to verify their behaviour. However, this characterization should be performed for all HT devices before measuring a single field component.

The additional advantage of the use of the E-field sensor with respect to the LED matrix is related to the achievable spatial resolution in the plane orthogonal to the E-field polarization direction. In fact, on that plane, the sensor moved by the robotic system can perform measurements with a resolution depending on the sensor’s radius and the spatial resolution of the robotic system. Considering these aspects, in the actual experimental set-up, a spatial resolution smaller than 5 mm is achievable in the mentioned plane. These aspects allow the E-field sensor to be more sensitive to changes in position or intensity of the EM focus than the LED matrix.

It is, however, to be noted that both experimental procedures, LED matrix and E-field sensor measurements, require control of the HT applicator, but the use of the E-field sensor also requires control of the robotic system for its movement, and control of the measurement instrumentation. Consequently, its use should be verified in terms of applicability in a clinical setting.

Finally, while the LED matrix can be used exclusively for qualitative measurements of the E-field distribution, the E-field sensor can undergo a calibration procedure that would make it capable of quantitative E-field measurements. Indeed, as a future development, a calibration procedure will be needed to link the sensor’s output voltage to the electric field radiated by the applicators. To this end, structures such as rectangular waveguides and TEM cells, partially filled with lossy liquids, have been proposed for calibrating electric field sensors for low-power electromagnetic compatibility applications (e.g., mobile communications) [23]. However, these procedures are equally applicable to the calibration of electric field sensors intended for radiative hyperthermia. In such cases, rectangular waveguides and TEM cells can be specifically designed to operate at HT frequencies and to accommodate the higher input powers typical of hyperthermia treatments. Important changes of the calibration procedure of the proposed sensor with respect to the sensors for mobile communication electromagnetic characterization are related to power generation and power measurement. When calibrating, it will be crucial to consider the working regime of the sensor depending on the input signal strength. Indeed, the sensor’s working regime must be well characterized and considered in the definition of the calibration factor.

The use of the isotropic sensor to characterize other commercial radiative HT devices is also a future development of this work. In addition, the characterization and optimization of the sensor extended to lower frequencies (around 10 MHz) could enable its use in verifying the electric field distribution and polarization for capacitive hyperthermia devices.

## Figures and Tables

**Table 1:** Short dipole condition in the vacuum.

		$\frac{l}{\lambda_{0}}$		
Dipole Length	70 MHz	100 MHz	434 MHz	915 MHz
2 cm	0.005	0.007	0.029	0.061
3 cm	0.007	0.010	0.043	0.091
4 cm	0.009	0.013	0.058	0.122
5 cm	0.012	0.017	0.072	0.152
6 cm	0.014	0.020	0.087	0.183

**Table 2:** Short dipole condition of the sensors immersed in the resin.

		$\frac{l}{\lambda_{r}}$		
Dipole Length	70 MHz	100 MHz	434 MHz	915 MHz
2 cm	0.0081	0.0115	0.0501	0.1057
3 cm	0.0121	0.0173	0.0752	0.1585
4 cm	0.0162	0.0231	0.1002	0.2113
5 cm	0.0202	0.0289	0.1253	0.2641
6 cm	0.0242	0.0346	0.1503	0.3170

**Table 3:** Effective lengths of the dipoles at 70 MHz, 100 MHz, 434 MHz, and 915 MHz.

		$h_{e}$		
Dipole Length	70 MHz	100 MHz	434 MHz	915 MHz
2 cm	0.90 cm	1.00 cm	1.00 cm	1.30 cm
3 cm	1.40 cm	1.70 cm	1.60 cm	\
4 cm	1.80 cm	2.10 cm	2.40 cm	\
5 cm	2.30 cm	2.70 cm	\	\
6 cm	2.80 cm	3.30 cm	\	\

## Data Availability

The original contributions presented in this study are included in the article. Further inquiries can be directed to the corresponding author.
